# Changes in body mass index, leptin and adiponectin in Japanese children during a three-year follow-up period: a population-based cohort study

**DOI:** 10.1186/1475-2840-8-30

**Published:** 2009-06-03

**Authors:** Rimei Nishimura, Hironari Sano, Toru Matsudaira, Aya Morimoto, Yumi Miyashita, Takako Shirasawa, Akatsuki Kokaze, Naoko Tajima

**Affiliations:** 1 Division of Diabetes, Metabolism and Endocrinology, Department of Internal Medicine, Jikei University School of Medicine, Tokyo, Japan; 2 Graduate School of Public Health, University of Pittsburgh, Pittsburgh, PA, USA; 3 Department of Public Health, Showa University School of Medicine, Tokyo, Japan

## Abstract

**Objective:**

The study examined changes in and relationship between body mass index (BMI), leptin and adiponectin levels over a 3-year period in a pediatric population-based cohort.

**Study design:**

A 3-year prospective cohort study of 268 boys and 251 girls aged 9–10 in Ina, Saitama, Japan.

**Results:**

Median body mass index (BMI) significantly increased from baseline (age 9–10) to follow up (age 12–13) in boys from 17.1 to 18.3 kg/m^2 ^(*P *< 0.001) and in girls from 16.5 to 18.5 kg/m^2 ^(*P *< 0.001), respectively. Adiponectin values significantly decreased from baseline to follow up in boys (13.5 to 8.9 μg/ml, respectively) (*P *< 0.001) and in girls (12.4 to 9.5 μg/ml, respectively) (*P *< 0.001). Leptin values at follow up significantly decreased from baseline in boys (4.9 to 2.3 ng/dl, respectively) (*P *< 0.001) and also in girls (5.3 to 5.1 ng/dl, respectively) (*P *= 0.049).

A relatively strong correlation was seen in BMI (Spearman's correlation coefficient, *r *= 0.864, *P *< 0.001 in boys; *r *= 0.873, *P *< 0.001 in girls), adiponectin (*r *= 0.705, *P *< 0.001 in boys; *r *= 0.695, *P *< 0.001 in girls), and leptin (*r *= 0.449, *P *< 0.001 in boys; *r *= 0.610, *P *< 0.001 in girls) before and after the three-year period.

The ratio of follow up to baseline BMI was negatively correlated with that for adiponectin (*r *= -0.224, *P *< 0.001 in boys; *r *= -0.165, *P *= 0.001 in girls) and positively correlated with that for leptin (*r *= 0.518, *P *< 0.001 in boys; *r *= 0.609, *P *< 0.001 in girls).

**Conclusion:**

This study demonstrated that baseline adiponectin, leptin and BMI values measured at ages 9–10 correlated with those measured three years later. However, adiponectin values decreased and leptin values increased in those subjects whose BMI increased during over this period.

## Introduction

The prevalence of pediatric obesity is rising sharply throughout the world, with marked increases also seen in Asia, including Japan [1-3]. While some studies have shown childhood obesity to be a risk factor for the development of adult obesity [4-6] as has low birth weight [7]. Another study has reported that less than half of the pediatric obese cases go on to become obese adults [8]. Therefore there is no clear consensus to the role of pediatric obesity in the development of adult obesity and no clear answers to questions such as what is the age of onset of pediatric obesity that leads to adult cardiovascular disease (CAD), and when intervention should be in initiated in obese children in order to prevent CAD.

Whitaker RC et al. [4] reported that the risk of childhood obesity progressing to adult obesity rises markedly after the age of 10. In Finland, an intervention study was conducted in which the intake of lipids was restricted, beginning at 7 months after birth. Results of this study demonstrated a significant difference in the incidence of obesity between the control and intervention groups after the age of 8 [9,10]. Another report has demonstrated that adult CAD patients tended to have low birth weight, with a lower-than-average BMI up to age 2, with a subsequent sharp increase in body weight before age 11, with the authors citing this growth pattern as a risk factor [7].

In our study, we focused on the 3-year-period corresponding to the ages during which the onset of obesity is most likely to lead to either adult obesity or CAD [4,7,10]. Within a specific region we were able to target almost all the children in this age group, studying changes in their body mass index (BMI), adiponectin and leptin levels, as well as the relationship between these values during the 3-year period to investigate any correlation between changes in these parameters and the development of obesity.

## Methods

This study was conducted as part of a pediatric health promotion program [11-14] initiated in 1994 in Ina, Saitama Prefecture, Japan. Ina is approximately 30 km to the north of Tokyo with a population of 35,000 comprising both farmers and residents who commute to work in Tokyo. In addition to annual national health checkups performed in accordance with the School Health Law, Ina has a unique health-promotion program in place. Of the fourth and seventh graders who had undergone regular health checkups, those who volunteered to take part in the program underwent blood and physical examinations.

The subjects of this study were children aged 9–10 who, in September 1999 or 2000, were all in the fourth grade in 3 elementary schools in town, and who agreed to participate in the study. A total of 304 boys and 282 girls took part in the study's initial survey (100% registration rate). A follow-up survey was conducted when the subjects reached the seventh grade (ages 12–13).

At the time of the initial and follow-up surveys, the subjects underwent blood tests including leptin and adiponectin measurements and physical measurements that included height and weight.

Adiponectin was measured in accordance with a report by Arita et al using commercially available ELISA kits (Otsuka Pharmaceutical Co., Ltd., Japan) with intra- and inter-assay coefficients of variation below 10% [15]. Leptin was measured using commercially available RIA kits (Linco Research Inc.) with intra- and inter-assay coefficients of variation below 10% [16]. BMI was used as an index for obesity status.

All values were expressed as median and interquartile range (IQR). The Kruskal-Wallis test was used to evaluate statistical significance.

Baseline values for each of the variables examined (leptin, adiponectin and BMI) were recorded when the subjects were 9–10 years old. These values were again measured three years later when the children were aged 12–13 (see Table 1). The ratio of the baseline compared to follow-up values were examined for each test variable. Spearman's correlation coefficient was used to evaluate any correlation between these values. Statistical analyses were made using the SPSS program.

**Table 1:** The median (interquartile range) for body mass index (BMI), adiponectin (μg/ml) and leptin (ng/dl) in the study participants at ages 9–10 and 12–13

Age group	9–10-year-old			12–13-year-old		
		
(n)	Boys (268)	Girls (251)	Total (519)	Boys	Girls	Total
BMI (kg/m^2^)	17.1 (15.7–18.7)	16.5 (15.2–18.3)	16.7 (15.4–18.7)	18.3** (16.9–19.8)	18.5** (17.0–20.3)	18.4** (17.0–20.1)
Adiponectin (μg/ml)	13.5 (9.6–18.2)	12.4 (9.5–16.5)	12.9 (9.6–17.5)	8.9** (6.9–11.3)	9.5** (7.4–11.8)	9.3** (7.2–11.6)
Leptin (ng/dl)	4.9 (3.2–7.8)	5.3 (3.8–9.2)	5.0 (3.5–8.5)	2.3** (1.6–3.6)	5.1* (3.6–7.9)	3.5** (2.1–6.0)

* *P *< 0.05, ** *P *< 0.001, vs. values measured at ages 9–10 years

The study protocol was approved by two independent institutional review boards (IRB) at Jikei University School of Medicine and Showa University School of Medicine. Written informed consent was obtained from all study participants and their guardians.

## Results

### Follow-up rates

Of the 586 children, follow-up physical and laboratory examination was performed on 519 subjects three years later (follow-up rate, 88.6%). Relocation was the major reasons that children were lost to follow-up. There was no statistical difference in baseline values between those available for follow-up and those lost to follow-up in gender (*P *= 0.512) and BMI (*P *= 0.958).

### Values measured at ages 9–10 and at ages 12–13

Body mass index (BMI) was significantly increased and adiponectin and leptin significantly decreased among study participants for both genders before and after the three-year follow-up. The mean baseline BMI value (kg/m^2^) for boys was 17.1 (15.7–18.7) increasing to 18.3 (16.9–19.8) at follow up (*P *< 0.001), and 16.5 (15.2–18.3) increasing to 18.5 (17.0–20.3) in girls (*P *< 0.001) respectively. The mean baseline adiponectin value (μg/ml) in boys dropped from 13.5 (9.6–18.2) to 8.9 (6.9–11.3) at follow up (*P *< 0.001), and dropped from 12.4 (9.5–16.5) to 9.5 (7.4–11.8) in girls (*P *< 0.001) respectively. The mean baseline leptin value (ng/dl) dropped from 4.9 (3.2–7.8) to 2.3 (1.6–3.6) in boys (*P *< 0.001) compared to a respective drop from 5.3 (3.8–9.2) to 5.1 (3.6–7.9) in girls (*P *= 0.049).

### Correlation analysis

**Figure 1:**
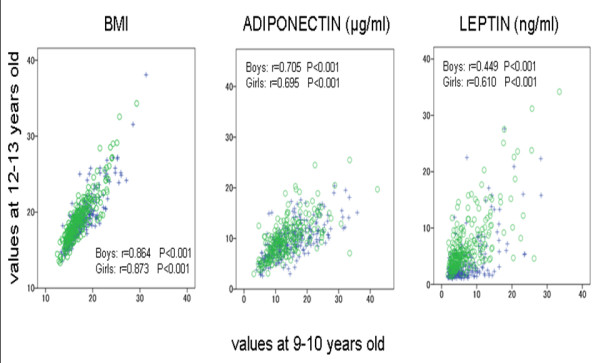
**Correlation in BMI, adiponectin and leptin between their values at ages 9 – 10 and their values measured at 12 – 13 by gender in the 519 children living in the town of Ina**. Boys (+): n = 268 Girls (○): n = 251. r: Spearman's rank correlation coefficients.

Baseline BMI, adiponectin and leptin values measured at ages 9–10 years demonstrated a significant positive correlation with those measured three year later at follow up: BMI (boys, *r *= 0.864, *P *< 0.001; girls, *r *= 0.873, *P *< 0.001); adiponectin (boys, *r *= 0.705, *P *< 0.001; girls, *r *= 0.695, *P *< 0.001), and leptin (boys, *r *= 0.449, *P *< 0.001; girls, *r *= 0.610, *P *< 0.001) (Figure 1).

**Figure 2:**
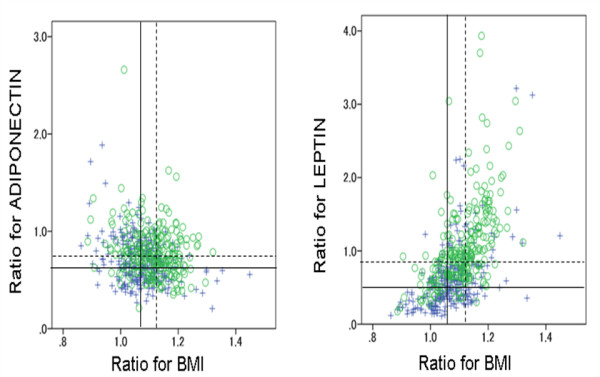
**Ratio of the values for BMI, adiponectin and leptin measured at ages 9 – 10 to those measured at 12 – 13, and their correlation by gender in the 519 children living in the town of Ina**. Boys (+): n = 268, Girls (○): n = 251. r: Spearmans' correlation coefficients. Ratio: values measured at ages 12–13/values measured at ages 9–10. Straight line, medians for boys; dotted line, medians for girls. Adiponectin: r = -0.224, P < 0.001 in boys, r = -0.165, P = 0.001 in girls. Leptin: r = 0.518, P < 0.001 in boys, r = 0.609, P < 0.001 in girls.

A negative correlation was found for the ratios of follow up to baseline adiponectin with that for BMI in both males and females (boys, *r *= -0.224, *P *< 0.001; girls, *r *= -0.165, *P *= 0.001). In contrast a positively correlation was reported for the follow up to baseline leptin ratio with that for BMI in both sexes (boys, *r *= 0.518, *P *< 0.001; girls, *r *= 0.609, *P *< 0.001) (Figure 2).

## Discussion

It is generally believed that obese children will progress to become obese adults [4-7] with reports demonstrating that the greater the age at which a child is obese, the greater the link to adult obesity [4,5,17,18]. In addition, the risk of child obesity progressing to adult obesity increases especially after the age of 10 [4]. Thus, in this study, we focused on the 3-year period between the ages of 9–10 and 12–13 representing the greatest risk group and investigated changes in BMI, leptin, and adiponectin.

This study targeted all eligible children living in a typical Japanese town with little population movement. This study was distinctive in that it prospectively followed changes in adipokines in a childhood population-based cohort over a 3-year period. The study results demonstrated that adiponectin, leptin and BMI values measured at baseline (9–10 years) correlated with those measured three years later. Therefore, these three variables measured at ages 9–10 were predictive for results obtained three years later, suggesting that in this age group these variables were likely to be genetically determined. However, adiponectin values were relatively decreased and leptin values relatively increased in children whose BMI increased during the 3-year period. Of note, Cambuli VM et al. reported that adiponectin and leptin were positive metabolic outcome markers following lifestyle intervention in overweight and obese children [19].

The current study showed that adiponectin values measured at ages 12–13 were lower than at ages 9–10. Similar findings have been reported in Germany and in USA, showing that adiponectin values in post-pubertal children are lower than in pre-pubertal children, especially in boys, which is explained by the elevated serum androgen levels in the former [20,21]. Other reports of age-related effects on adiponectin show that the adiponectin value drops in a 2-year-old to a greater extent than in a 1-year-old [22], and that the adiponectin values do not change in women aged 18 to 80 [23].

This study also demonstrated that the leptin values at ages 12–13 were lower than those at ages 9–10, especially in boys. Leptin values, which correlate with the amount of body fat present, are reported to increase in girls and decrease in boys after Tanner stage 2 as the pubertal developments proceeds [24]. Another study conducted in 2- to 5-year-old Tsimané children showed that the correlation between body fat and leptin increases markedly with advancing age in females [25]. The lower levels of leptin in boys may partly be explained by the suppressive effect of androgens in the boys [24]. However, our study showed no age-related increase in leptin values in girls.

The current study examined changes in BMI and adipocytokines in Japanese children. Differences in adipocytokines between ethnic groups have been demonstrated in previous reports in the literature [26,27]. Therefore, these ethnic differences should be taken into account when the results of this study are used for international comparisons.

Limitations of this study include the lack of confirmation of pubertal stage (Tanner's stages) of all subjects, as this would have been useful to correlate with the data obtained; this was an observational study covering a specific 3-year period and therefore the values obtained for the metabolic markers may not necessarily be linked directly to adult obesity. This study examined the relationship between the changes in test variables and obesity, and did not take the subjects' lifestyles into consideration. Therefore, evaluation of lifestyle factors is an issue that needs to be addressed in the future.

In conclusion we demonstrated in a Japanese population-based childhood cohort that baseline adiponectin, leptin and BMI values measured at ages 9–10 correlated with those measured three years later. In addition, those children with an increase in BMI over this period were also found to have decreased adiponectin and increased leptin values. These results suggest that lifestyle intervention in obese children should be initiated between ages 9 and 12.

It is hoped that continued follow-up of the study participants will help identify potential methods of preventing the onset of visceral obesity and CAD in adulthood.

## Competing interests

The authors declare that they have no competing interests.

## Authors' contributions

RN and NT participated in the design of the study and performed the statistical analyses. HS, TM, AM, YM, TS, and AK participated in the coordination of the study. RN helped to draft the manuscript. All authors have read and approved the final manuscript.
